# Effect of Respiratory Failure on Peripheral and Organ Perfusion Markers in Severe COVID-19: A Prospective Cohort Study

**DOI:** 10.3390/jcm13020469

**Published:** 2024-01-15

**Authors:** Mateusz Gutowski, Jakub Klimkiewicz, Bartosz Rustecki, Andrzej Michałowski, Kamil Paryż, Arkadiusz Lubas

**Affiliations:** 1 Department of Anesthesiology and Intensive Care, Military Institute of Medicine-National Research Institute, 04-141 Warsaw, Poland; jklimkiewicz@wim.mil.pl (J.K.); brustecki@wim.mil.pl (B.R.); amichalowski@wim.mil.pl (A.M.); kparyz@wim.mil.pl (K.P.); 2 Department of Internal Diseases, Nephrology and Dialysis, Military Institute of Medicine-National Research Institute, 04-141 Warsaw, Poland; alubas@wim.mil.pl

**Keywords:** perfusion, microcirculation, DTPM, capillary refill time, infrared camera, COVID-19, oxygenation ratio, ICU

## Abstract

Microvascular dysfunction and inflammation caused by COVID-19 disrupt organ function. The study aimed to investigate the association between the severity of SARS-CoV-2 pneumonia and peripheral and organ perfusion as a consequence of altered microcirculation. A total of 116 patients hospitalized due to severe COVID-19 were enrolled in the study. On admission, the patients underwent a Capillary Refill Time (CRT) examination, finger oxygen saturation measurement, thermal imaging of the hand (FIT), and a kidney Doppler ultrasound. Medical data were collected from the medical history. From the evaluated perfusion parameters, only renal cortex perfusion (RCP) was substantially correlated with the CT score (*p* < 0.010). The peripheral perfusion parameters of Sat., FIT, CRT, and RCP correlated with the ARDS stages (*p* = 0.0021; *p* = 0.038; *p* < 0.0006; *p* < 0.0002, respectively). The Oxygenation Ratio value (*p* < 0.001) was significantly associated with all the perfusion parameters (saturation, CRT, FIT, and RCP) in the multivariable regression analysis model. According to the stepwise retrograde regression analysis, RCP was an independent parameter linked with the Oxygenation Ratio (*p* < 0.001). Severe COVID-19 can result in microvascular dysfunction influencing peripheral and organ perfusion, which can be measured with various methods. The staging of COVID-19 assessed by CT and the Oxygenation Ratio correlates with RCP, CRT, FIT, and oxygen saturation.

## 1. Introduction

Adults with COVID-19 can manifest a mild infection with no symptoms to severe infection with rapid progression to respiratory or multi-organ failure [1]. Sufficient organ perfusion is one of the conditions for its proper function. Impaired perfusion in critical condition can lead to organ ischemia, which is associated with poor prognosis [2,3]. While measuring the cardiac output (CO) and other hemodynamic parameters provides information vital to the clinical management of the critically ill, one can also argue that the accurate status of oxygen delivery and/or the adequacy of tissue perfusion can be similarly useful. 

There are many methods to measure cardiac output: pulmonary artery catheter, echocardiography, transpulmonary thermodilution, esophageal Doppler, minimally invasive or noninvasive pulse wave analysis. Numerous studies have documented the superiority of dynamic over static variables to predict fluid responsiveness such as stroke volume variation or pulse pressure variation or passive leg raising or variation in inferior vena cava [4,5,6,7].

Shock is a clinical diagnosis based on the signs and symptoms of inadequate tissue perfusion. Normal or even high values of CO and MAP could still be insufficient for high metabolic demand, often observed in the critically ill [8]. Damage mechanisms to microcirculation include but are not limited to direct insult caused by viruses, damage to endothelial cells and thromboinflammation [9], the dysregulation of immune reactions and hyperinflammation caused by the inhibition of interferon signaling by viruses, the lymphodepletion of T cells and the production of proinflammatory cytokines, especially IL-6 and TNF [10].

Several observations suggest a relationship between the onset of hemostasis disorders and the severity of respiratory failure. Also, mortality in patients with COVID-19 is higher when abnormalities are observed in hemostasis laboratory tests. The presence of hemostasis impairment is evidenced by elevated levels of D-Dimers and fibrinogen, prolonged prothrombin time and thrombocytopenia [11,12]. Patients with COVID-19 may experience excessive thrombin production, inhibited fibrinolysis, complement pathways activation, thromboinflammation, leading to microthrombi and microvascular dysfunctions [11,12,13,14,15]. Generalized endothelial dysfunction is observed in sepsis and viral, bacterial, and fungal septic shock [16]. Diffuse microcirculatory damage due to endothelial damage is frequently observed in the severe course of COVID-19 [13,17]. This was confirmed by observations of capillary congestion found in the lungs of the deceased [18]. However, these changes are not only limited to the lungs. Microcirculatory disturbances also affect the brain, heart, gastrointestinal tract, kidneys, and liver [9,13,19,20,21,22,23,24]. As changes in peripheral perfusion seem to be of critical importance, it should be monitored.

The Capillary Refill Time (CRT) is a simple, widely available test assessing microcirculation [25]. Unfortunately, this test carries a high risk of variability depending on the provider [26]. Other instrumental methods assessing peripheral microcirculation seem to have less variability; most commonly, pulse oximetry and the thermographic evaluation of the hand [27,28]. Available scientific data prove that CRT is useful among patients in shock, where disequilibrium between vasodilators, vasoconstrictors, and endothelial cells leads to severe peripheral flow disturbances. Signs of compromised peripheral perfusion detected by CRT are early markers of inadequate organ perfusion among patients in shock [29,30,31]. CRT performed within 24 h of Intensive Care Unit (ICU) admission enables the identification of patients who will develop elevated blood lactate levels and severe organ failure [32]. A thermal imaging camera can also be used to monitor peripheral perfusion. A decrease in peripheral temperature may indicate peripheral hypoperfusion. Experimental animal studies have shown a significant relationship between the decrease in limb temperature, blood pressure, heart rate, and cardiac output [33]. 

Acute kidney injury (AKI), isolated or accompanied by the insufficiency of other systems, is a common complication of septic shock [34]. AKI is independently associated with increased mortality in patients with septic shock [35]. In addition to inadequate perfusion, damage to the microcirculation caused by sepsis, increased leukocyte adhesion, rheological disorders, and changes in glycocalyx play an important role in the development of AKI [36,37,38]. 

In recent years, the non-invasive method of dynamic tissue perfusion measurement (DTPM), based on ultrasound, was introduced and successfully used to assess blood perfusion in the renal cortex [39,40]. Renal cortical perfusion (RCP) estimated by DTPM is quantified by the volume of blood flowing through the studied area (ROI—region of interest) over a specified period of time, which is equivalent to organ perfusion [40]. This non-invasive and readily available test proved its efficacy [41,42] in various clinical scenarios [43,44,45,46].

Our primary goal was to better understand how COVID-19 affects microcirculation in the form of peripheral and organ perfusion. We wanted to investigate the potential relationships between generalized COVID and microcirculation. 

For this purpose, we assessed the severity of COVID based on lung involvement evaluated by CT scan. The clinical assessment was based on the oxygenation index. Peripheral perfusion was estimated using CRT, peripheral oxygen saturation, and a thermographic image of the hand. Organ perfusion, represented by the kidney, was assessed using the DTPM method.

This study aimed to test the usefulness of DTPM and different non-invasive peripheral perfusion assessment methods in various stages of severe SARS-CoV-2 pneumonia.

## 2. Materials and Methods

A prospective cohort study was conducted in 2021–2022 at the COVID-19 Temporary Hospital of the Military Institute of Medicine, Warsaw, Poland. Every admitted patient suffered from a confirmed, severe form of COVID-19, including hypoxemia with the need for oxygen administration: standard nasal catheter, oxygen mask, reservoir mask, high-flow nasal oxygen therapy (HFNOT), non-invasive ventilation (NIV), mechanical ventilation.

Severe COVID was recognized in individuals who had SpO_2_ < 94% of room air at sea level, a ratio of arterial partial pressure of oxygen to fraction of inspired oxygen (PaO_2_/FiO_2_) < 300 mm Hg, a respiratory rate > 30 breaths/min, or lung infiltrates > 50% [1]. Hypoxemia was quantified by the Berlin criteria of ARDS PaO_2_/FiO_2_ 0: >300; 1-mild: 300 > x > 200; 2-moderate: 200 > x > 100; 3-severe: >100.

The study was conducted in accordance with the Helsinki Declaration and approved by the Institutional Bioethics Committee on 19 May 2021 (NR22/WIM/2021). Study inclusion criteria encompass confirmed diagnosis of COVID-19, age under 70 years, and patient’s ability to consent to participate. The only exclusion criterion was an inability to provide written informed consent or a later signed withdrawal consent.

All medical data were taken, and diagnostic examinations were performed on the first or second day of hospitalization. The perfusion study consisted of performing an image of the hand taken by an infrared camera (FIT—finger infrared thermography), saturation measurement (pulse oximetry), Capillary Refill Time (CRT) and Doppler ultrasound of the kidney. Medical history and comorbidities were obtained during the medical interview and with the revision of medical records. On admission, the patients underwent High-Resolution Computed Tomography of the lungs. Patients underwent biochemical and blood gas tests depending on the severity of the disease and current needs.

### 2.1. Fingertips Thermography

**Figure 1:**
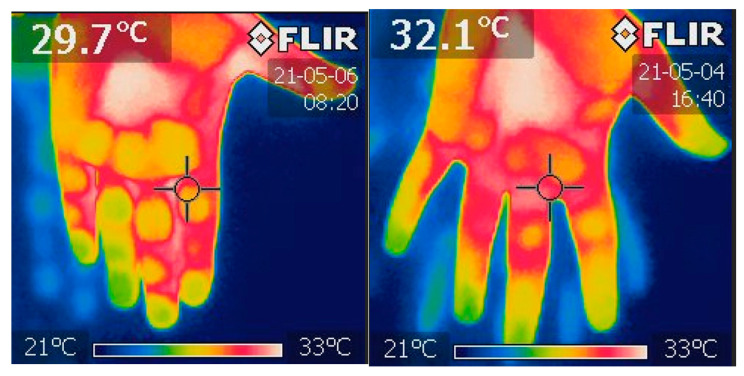
This is a thermal image of hands taken using infrared FLIR i7 camera. Own materials. The temperature of each distal phalanx was then examined independently using the ThermaBase application. More analysis was performed on the data derived from the mean temperature calculated for each phalange.

During every examination, a thermal image of the hands was taken using an infrared FLIR i7 camera (FLIR) (Figure 1). The photo was taken by pointing a hand at around 0.5 m. The camera was turned on in the patient’s room a quarter of an hour before performing an image for proper camera calibration. The infrared temperature count of distal phalanges was analyzed using Image ThermaBase EU 3.0.0.59 (WIM-PIB, Poland) [47]. The result is the mean temperature value of all phalanges.

### 2.2. Pulse Oximetry

A pulse oximetry examination was conducted with the use of a Sanity Duo Control device (Albert Hohlkörper GmbH & Co. KG, Hemer, Germany). The test was performed on the finger of the right and left hand for about one minute. The result was the average from both measurements.

### 2.3. Capillary Refill Time

CRT was measured by firm pressure applied to both hands’ ventral surface of the II and IV finger distal phalanx. Pressure was applied until the skin went blank, and it was held in place for five seconds. The time was measured by the examiner using a stopper. Then, the time until the skin’s natural tone has returned was noted. The mean of all measurements was used to calculate the result [48].

### 2.4. Doppler Ultrasound

**Figure 2:**
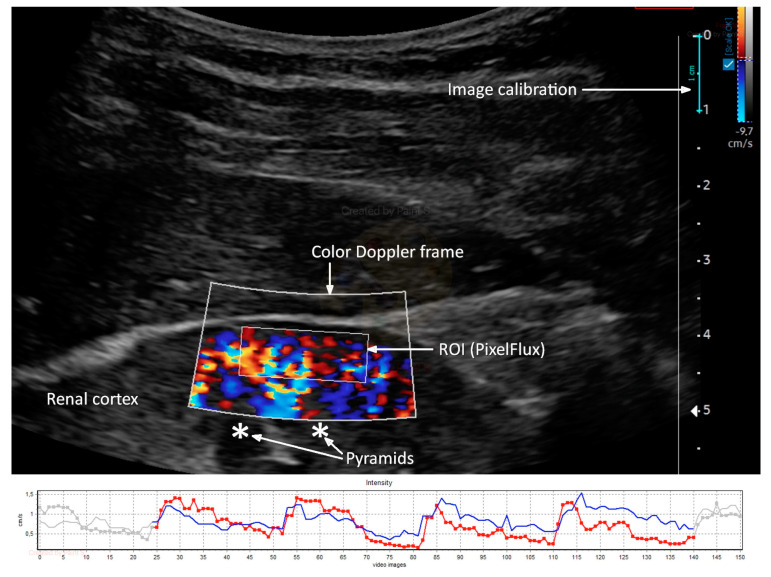
Ultrasound color Doppler image of renal cortex perfusion, analyzed with PixelFlux software (upper part) with a graphical presentation of arterial (red) and venous (blue) flow intensity measurement (lower part). Own materials.

Renal Doppler ultrasound was conducted using an ultrasound machine (Samsung HS40, Suwon, Republic of Korea) equipped with a CA2-8AD (2–8 MHz) convex transducer. A color Doppler frame was positioned on the longitudinal portion of the right kidney cortex’s mid-segment to visualize cortical vessels directly heading the transducer. The color Doppler gain was set constantly and never changed. However, the flow velocity scale was set at 9.7 cm/s, which could be carefully adjusted to improve flow visualization and artifact prevention. To stabilize the image, the patient was instructed to stop breathing in the middle of the exhalation. Two to three DICOM-formatted movie files with the color Doppler picture sequences lasting three to five complete heart cycles were recorded. An external medical device was used to calculate Renal Cortical Perfusion (RCP) as the average arterial and venous flow intensity throughout the entire cortex in the studied section (PixelFlux, Chameleon Software, Leipzig, Germany). The region of interest (ROI) was placed on the renal cortex, free of focal abnormalities, between the kidney surface and the outer border of the medullary pyramids, and inside the color Doppler frame (Figure 2).

### 2.5. High-Resolution Computed Tomography

The computed tomography (CT) severity score developed by Francone et al. [49] was used to evaluate the degree of lung involvement. Every lobe was assessed separately, with a minimum rank ranging from zero points (no involvement) to five points (more than 75 percent of parenchyma involved in the investigated lobe). A healthy individual’s five-lobe CT scan will result in a sum of 0 points, whereas a maximum of 25 points indicates that more than 75% of each lobe’s parenchyma is involved. Results with 18 or more points were found to be substantial predictors of mortality [49].

### 2.6. Oxygenation Ratio—PaO_2_/FiO_2_

The Oxygenation Ratio was defined as the ratio of the partial pressure of oxygen in the blood (PaO_2_) in millimeters of mercury and the fraction of oxygen in the inhaled air (FiO_2_)—the PaO_2_/FiO_2_ ratio. The Oxygenation Ratio is commonly used in the Berlin Criteria of ARDS to assess the severity of respiratory failure [50]. Analyzing the investigated population according to the severity of the course of ARDS according to the Berlin criteria, we defined the ARDS stages based on PaO_2_/FiO_2_ as 0: >300; 1-mild: 300 > x > 200; 2-moderate: 200 > x > 100; 3-severe: >100.

### 2.7. Statistical Analysis

Descriptive statistics were presented as means with standard deviation or median with interquartile range. Nominal variables were recorded as numbers with the percentage occurrence. To check the normality of the variable distribution, the Shapiro–Wilk test was performed. Pearson’s and Spearman’s tests were conducted for correlation analyses in the dependency of distribution normality. Point-biserial correlation analysis was performed to test the association between dichotomic and continuous variables. Differences between more than two groups were checked using analysis of variance (ANOVA) for normally distributed variables; otherwise, they were checked with Kruskal–Wallis’s test.

Multivariable regression analysis with a stepwise retrograde option was conducted to estimate factors substantially associated with the dependent variable. Double-sided *p* < 0.05 was considered significant. Statistical analysis was performed using Tibco Statistica software v. 13.3 (StatSoft Polska Sp z o. o., Cracow, Poland).

## 3. Results

One hundred and sixteen patients were enrolled in the study (46 F, 70 M, age 61.8 ± 15.0). Demographic data and medical history are presented in Table 1.

The results of the performed tests in the study population are presented in Table 2.

Significant correlations between performed tests are shown in Table 3.

Saturation correlated significantly only with creatinine and urea, whereas the CRT was associated with the Oxygenation Ratio, HR, serum albumin, and urea (Table 3). In contrast, renal cortex perfusion (RCP) was significantly correlated with the Oxygenation Ratio (*p* < 0.0001), serum concentration of lactate dehydrogenase, urea, and as the only perfusion variable with a CT score (*p* = 0.010). Of particular interest are the correlations linking the peripheral and organ perfusion parameters with COVID-19 disease severity based on the Oxygenation Ratio (Table 4).

The analysis of the differences between the ARDS stages and estimated variables is presented in Table 5. It turned out that the mean or median values of FIT (*p* = 0.038), CRT (*p* < 0.0006), RCP (*p* < 0.0002), saturation (*p* = 0.0021), as well as some biochemical data, especially urea (*p* = 0.016) and LDH (*p* < 0.00001), were significantly different between the ARDS stages. On the other hand, haemodynamic parameters such as MAP, SBP, and DBP (*p* = 0.683, *p* = 0.718, and *p* = 0.474, respectively) and biochemical parameters in the form of albumin (*p* = 0.175) and creatinine (*p* = 0.151) did not differentiate between the ARDS groups.

**Figure 3:**
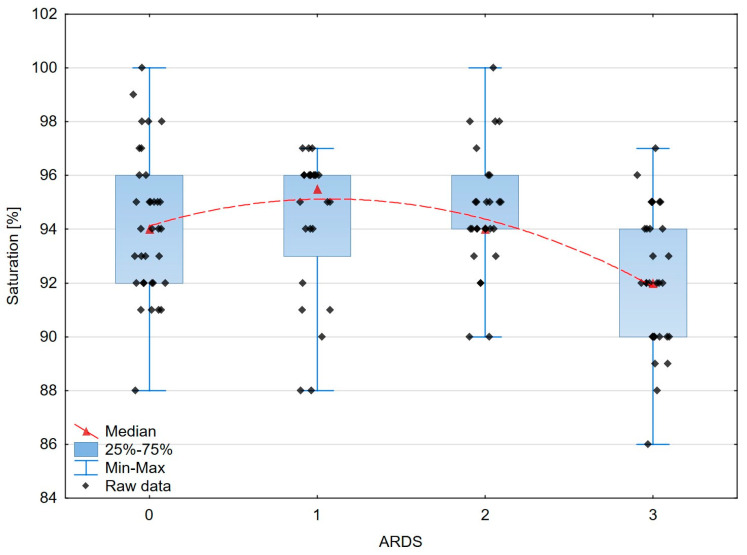
The box–whisker scatterplot expresses the relation between the saturation and the ARDS stages. Red dashed line—polynomial curve expressing the approximated correlation.

**Figure 4:**
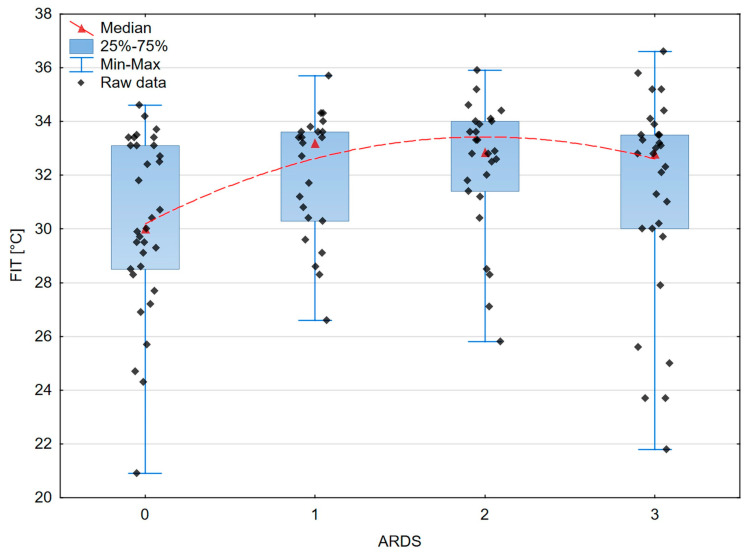
The box–whisker scatterplot expresses the relation between finger infrared thermography and the ARDS stages. Red dashed line—polynomial curve expressing the approximated correlation.

**Figure 5:**
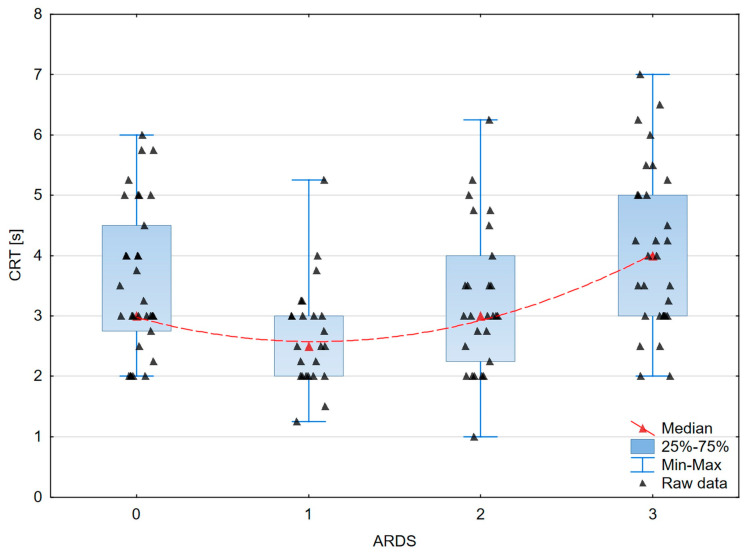
The box–whisker scatterplot expresses the relation between Capillary Refill Time and the ARDS stages. Red dashed line—polynomial curve expressing the approximated correlation.

**Figure 6:**
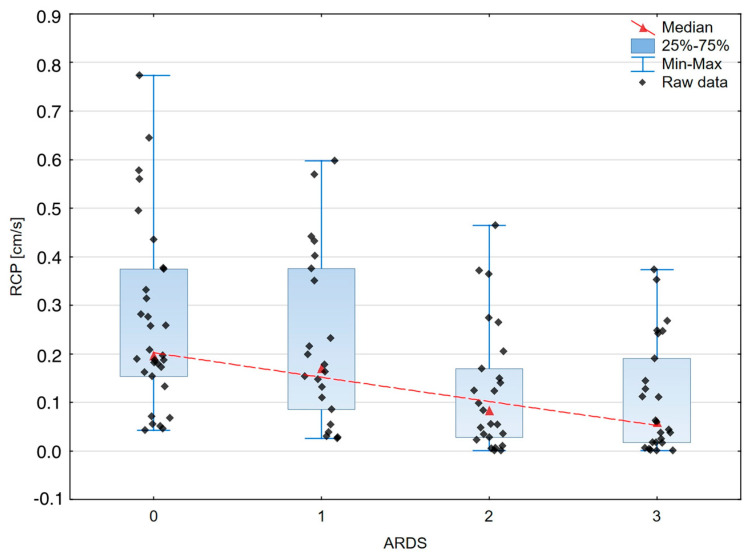
The box–whisker scatterplot expresses the relation between Renal Cortical Perfusion and the ARDS stages. Red dashed line—polynomial curve expressing the approximated correlation.

A graphical presentation of the peripheral and organ perfusion markers’ associations with the severity of ARDS is shown in Figure 3, Figure 4, Figure 5 and Figure 6.

In the model of multivariable regression analysis including perfusion parameters (saturation, CRT, FIT, RCP) all were significantly associated with the Oxygenation Ratio value (R^2^ = 0.264; *p* < 0.001) (Table 6). However, the stepwise retrograde regression analysis performed in the same model revealed that the RCP was the best independent parameter associated with the Oxygenation Ratio (R^2^ = 0.120; *p* < 0.001) (Table 7).

## 4. Discussion

COVID-19 is primarily a pulmonary disease, but broad data also suggest cardiac, hematologic, neurologic, renal, and thromboembolic events [51,52,53,54,55,56]. There are reports indicating endothelial damage caused by COVID-19 [13].

In this study, we investigated the relationship between perfusion disorders and the severity of COVID-19. Our work fills a gap in the literature, as no papers linking respiratory failure and perfusion disorders were studied with the methods we propose.

Microcirculation disorders during sepsis are visible in every organ: heart, intestine, liver, brain, or kidney [57].

In the case of the kidney, its function is closely linked to blood flow. The relationship between function and flow gives us research perspectives in the context of the assessment of biochemical parameters such as creatinine and urea. The numerous relationships between renal macro- and microcirculation and sepsis are perfectly described by Post et al. It is worth noting the pathophysiology of kidney function described in this article, including hypoxemia and the role of nitric oxide or reactive oxygen species, which are particularly interesting in the context of COVID [58].

In our study, we decided to evaluate the peripheral microcirculation with CRT, FIT, and saturation, whereas to assess the organ perfusion, we selected a kidney by examining it using Doppler ultrasound. We selected the oxygenation index and lung CT imaging to evaluate the severity of COVID-19.

The paper by Jakob et al., “ARDS. Tissue perfusion monitoring” from 2002, indicated that at that time, there were no appropriate methods of microcirculation monitoring [59].

Garrick et al. looked at micro- and macro-circulation endpoints in the fluid resuscitation of patients with sepsis. For the macrocirculation assessment, lactate, MAP, and CO were examined; the CRT was used to investigate microcirculation [60].

Our data show a statistically significant correlation between the CRT and Oxygenation Ratio (*p* < 0.03) and urea (*p* = 0.011). The microcirculation is regulated by flow pressure differences, muscle tension, and arteriole resistance [51]. It has been demonstrated that changes in the ratio of vasoconstrictors to vasodilators, proinflammatory markers, and microthrombi development lead to the decreased control of microcirculatory pressure and perfusion, which compromises tissue oxygenation in critically ill patients [52,53]. The CRT test is basic and widely available at the bedside. The Andromeda-Shock 1 study demonstrated that the CRT-based resuscitation of septic shock patients is as good as lactate-based [48]. Also, in the evaluation of fluid resuscitation in children with sepsis, the CRT has shown its usefulness [54]. In COVID itself, microvascular impairment measured by the CRT was described [3].

According to our results, the correlation of the fingertip temperature (FIT) with the severity of ARDS showed a trend to statistical significance (*p* = 0.054).

However, it appeared to be the independent factor connected with the Oxygenation Ratio value (*p* = 0.004). The analysis of the thermographic image also seems to be promising in septic shock [55]. The study by Amson et al. showed a core-to-skin gradient as a useful tool for predicting the 8-day mortality in septic shock patients [56].

Moreover, we found statistically significant associations concerning the relationship between the severity of ARDS and oxygen saturation measured in the finger (*p* = 0.012).

In the presented study, impaired kidney perfusion (RCP) correlates with the deterioration of gas exchange (Oxygenation Ratio; *p* < 0.000035 and CT score; *p* = 0.01).

Moreover, statistically significant associations concerning the relationship between the severity of ARDS and renal cortex perfusion, RCP, (*p* < 0.0001) were revealed. Nevertheless, according to the stepwise retrograde multivariable regression analysis, RCP was the best independent parameter linked to the Oxygenation Ratio.

Our results also show a correlation between the RCP and urea concentration (*p* = 0.031), which is consistent with the view of reduced renal flow and impaired renal function in sepsis [41,58]. Existing studies show a decrease in the blood flow through the cortex and medulla of the kidneys in patients with severe COVID-19, visible on magnetic resonance imaging [61].

Our results show a significant association between the severity of respiratory failure and the impairment of peripheral and organ perfusion, which seems to be consistent with the pathophysiology of the development of ARDS. The usefulness of DTPM developed by Scholbach was investigated in many clinical applications [39,40,41,43,44]. Lubas et al. proved its usefulness in the assessment of renal perfusion among patients with hypertension and glomerulonephritis. However, DTPM was never investigated in ARDS or septic patients.

Interesting conclusions also emerged from the quantitative analysis, potentially having an impact on our daily clinical practice.

It turns out that the peripheral perfusion parameters FIT (*p* = 0.038), CRT (*p* < 0.0006), RCP (*p* < 0.0002), and saturation (*p* = 0.0021) are better related to the severity of gas exchange than the pressure assessment, MAP, SBP, and DBP (*p* = 0.683, *p* = 0.718, and *p* = 0.474, respectively) (Table 5).

Huai-wu et al. showed that the Perfusion Index and the transcutaneous oxygen challenge test (OCT) (a test that evaluates the transcutaneous partial pressure of oxygen (PtcO_2_) in response to 100% oxygen respiration) are better predictors of mortality in sepsis than the macrocirculation parameter such as the central venous oxygen saturation (ScvO_2_) [62].

The presented study has some limitations. First, CRT and ultrasound examination are subjective, operator-related procedures. Second, due to the contagious nature of COVID-19, the need for personal protection equipment can influence performed examinations. One of the main limitations of this study is the variability between patients. We must bear in mind that 29% of the patients with COVID-19 did not meet the Berlin criteria of the ARDS (PaO_2_/FiO_2_ > 300); however, they meet the criteria for severe COVID due to oxygen therapy. We are aware that the 2012 Berlin Criteria, by definition, require a PEEP above 5 cmH_2_O. However, we rely on the latest guidelines from 2023, in which patients receiving HFNO are included as meeting the Berlin Criteria [50].

Moreover, due to the necessity of written informed consent, patients with consciousness disorders were not included. Nevertheless, the advantage of our study is its prospective and observational nature. The above research sheds interesting insights linking ARDS and microcirculation disorders together, a topic that requires further research.

## 5. Conclusions

The severe course of COVID-19 can influence peripheral and organ perfusion, which can be measured with various methods and can be the reason for multi-organ failure development. Capillary Refill Time, finger saturation, finger infrared thermography, and the renal cortex ultrasound dynamic tissue perfusion measurement are suitable for assessing peripheral and organ perfusion changes according to severe COVID-19 advancement. The variability of the renal cortex perfusion representing changes in the organ blood supply seems to be the best parameter expressing COVID-19-related acute lung injury. However, the use of renal cortical perfusion in assessing other COVID-19 manifestations or predicting the outcome of SARS-CoV-2 infection requires further studies.

## Figures and Tables

**Table 1:** Descriptive data, demography, diseases, and ARDS severity based on Oxygenation Ratio—PaO_2_/FiO_2._

Variable			N	%
Gender	Male		70	60.3
	Female		46	39.7
Ward Type	Intensive Care Unit		38	32.8
	High-Dependency Unit		78	67.2
Comorbidity	Malignancy		11	9.5
	Obesity		19	16.4
	Chronic Kidney Disease	Non-dialyzed	4	3.4
		Dialyzed	1	0.9
	Coronary Artery Disease		7	6.0
	Heart Failure		6	5.2
	Myocardial infarction		2	1.7
	CABG/Stent		7	6.0
	Atrial fibrillation	Chronic	7	6.0
		De novo	4	3.4
	Hypertension		33	28.4
	Diabetes	Non-insulin	14	12.1
		Insulin dependent	8	6.9
	Asthma		7	6.0
	Chronic Obstructive Pulmonary Disease		6	5.2
	Smoker		4	3.4
ARDS	PaO_2_/FiO_2_ > 300	No ARDS	34	29.3
	PaO_2_/FiO_2_ 300 to 200	MILD	24	20.7
	PaO_2_/FiO_2_ 200 to 100	MODERATE	27	23.3
	PaO_2_/FiO_2_ < 100	SEVERE	31	26.7

CABG—Coronary Artery Bypass Graft; FiO_2_—Fraction of Inspired Oxygen; PaO_2_—Pressure of Oxygen; Oxygenation Ratio—PaO_2_/FiO_2_.

**Table 2:** Results of performed tests in vital signs and biochemical parameters.

	Median (Mean)	IQR (±SD)
CT score [0/25]	16.0	10.0
FiO_2_ [%]	0.40	0.59
PaO_2_ [mmHg]	75.0	24.0
Oxygenation ratio	205.0	215.5
Saturation [%] *	(93.7)	(±2.7)
FIT [°C]	32.6	4.0
CRT [s]	3.0	1.8
RCP [cm/s]	0.149	0.224
MAP [mmHg]	94.3	15.0
SBP [mmHg]	130.0	21.0
DBP [mmHg] *	(78.4)	(±12.6)
HR [1/min]	84.0	21.5
Albumin [g/dL] *	(3.1)	(±0.4)
Creatinine [mg/dL]	0.90	0.40
Urea [mg/dL]	35.0	24.0
LDH [U/L]	408.5	244.0

*—variable with normal distribution. IQR—Interquartile Range; SD—Standard Deviation; CT score—based on Computer Tomography lung involvement severity score; FiO_2_—Fraction of Inspired Oxygen; PaO_2_—Pressure of Oxygen; Oxygenation Ratio—PaO_2_/FiO_2_; saturation—peripheral saturation of oxygen; CRT—Mean Value of Capillary Refill Time; RCP—Renal Cortical Perfusion; MAP—Mean Arterial Pressure; SBP—Systolic Blood Pressure; DBP—Diastolic Blood Pressure; HR—Heart Rate; LDH—Lactate dehydrogenase.

**Table 3:** Significant correlations between performed tests.

Variable	Correlation Coefficient—r	*p*-Value
Saturation and Creatinine	−0.194	0.038
Saturation and LDH	−0.314	0.002
CRT and Oxygenation Ratio	−0.202	0.030
CRT and HR	0.196	0.042
CRT and Albumin	−0.403	0.005
CRT and Urea	0.235	0.011
RCP and CT score	−0.275	0.010
RCP and Oxygenation Ratio	0.396	<0.0001
RCP and LDH	−0.221	0.049
RCP and Urea	−0.213	0.031

Saturation—peripheral saturation of oxygen; LDH—Lactate dehydrogenase; CRT—Mean Value of Capillary Refill Time; Oxygenation Ratio—PaO_2_/FiO_2_; HR—Heart Rate Beats/Minute; RCP—Renal Cortical Perfusion; CT score—based on Computer Tomography lung involvement severity score.

**Table 4:** Correlation analysis between perfusion parameters and ARDS severity.

Variable	Correlation Coefficient—r	*p*-Value
Saturation and ARDS severity	−0.232	0.012
FIT and ARDS severity	0.183	0.054
CRT and ARDS severity	0.173	0.064
RCP and ARDS severity	−0.426	<0.0001

FIT—Phalanx Temperature; CRT—Mean Value of Capillary Refill Time; RCP—Renal Cortical Perfusion.

**Table 5:** Comparison of perfusion, hemodynamic, biochemical variables in ARDS stages.

	ARDS								*p*-Value
	No ARDS PaO_2_/FiO_2_ > 300		1 MILD PaO_2_/FiO_2_ 300 to 200		2 MODERATE PaO_2_/FiO_2_ 300 to 200		3 SEVERE PaO_2_/FiO_2_ 300 to 200		
	Median (Mean)	IQR (±SD)	Median (Mean)	IQR (±SD)	Median (Mean)	IQR (±SD)	Median (Mean)	IQR (±SD)	
Saturation [%]	94	4	96	3	94	2	92	4.0	0.0021
FIT [°C]	30	4.6	33.2	3.3	32.9	2.6	32.8	3.5	0.038
CRT [s]	3.0	1.75	2.5	1	3	1.8	4.0	2.0	<0.0006
RCP [cm/s]	0.196	0.22	0.171	0.29	0.083	0.141	0.059	0.173	<0.0002
MAP [mmHg] *	(93.2)	(±12.9)	(95.2)	(±16.7)	(97.6)	(±14.4)	(95.5)	(±10.0)	0.683
SBP [mmHg]	128	18	126	27	130	20	130	20	0.718
DBP [mmHg] *	(76.4)	(±12.6)	(79.7)	(±11.6)	(81.0)	(±13.8)	(77.2)	(±12.1)	0.474
HR [1/min]	81	14	75	23	87	20	91	18	0.052
Albumin [g/dL]	3.4	1.05	3.55	0.6	3.2	0.3	3.1	0.4	0.175
Creatinine [mg/dL]	1	0.4	0.8	0.2	0.9	0.5	1	0.5	0.151
Urea [mg/dL]	34	24	27	17.5	38	19	45	35	0.016
LDH [U/L]	365.5	207.5	319	109	435	195	591.5	165	<0.00001

*—variable with normal distribution. ARDS—Acute Respiratory Distress Syndrome; IQR—Interquartile Range; SD—Standard Deviation; PaO_2_—Pressure of Oxygen; FiO_2_—Fraction of Inspired Oxygen; saturation—peripheral saturation of oxygen; FIT—finger infrared thermography; CRT—Mean Value of Capillary Refill Time; RCP—Renal Cortical Perfusion; MAP—Mean Arterial Pressure; SBP—Systolic Blood Pressure; DBP—Diastolic Blood Pressure; HR—Heart Rate; LDH—Lactate dehydrogenase.

**Table 6:** Results of Oxygenation Ratio multivariable regression analysis.

	Beta	Beta Standard Error	*p*-Significance
Saturation [%]	0.225	0.088	0.013
FIT [°C]	−0.293	0.099	0.004
CRT [s]	−0.247	0.099	0.015
RCP [cm/s]	0.318	0.088	<0.001

Saturation—peripheral saturation of oxygen; CRT—Capillary Refill Time; FIT—finger infrared thermography; RCP—Renal Cortical Perfusion.

**Table 7:** Results of Oxygenation Ratio prediction modeling according to RCP values.

RCP (cm/s)	Oxygenation Ratio	95% CI
0.770	363	281–444
0.185	219	196–241
0.001	173	140–206

RCP—Renal Cortical Perfusion; CI—Confidence Interval.

## Data Availability

The dataset is maintained by the authors and available on request.
